# Efficient Carbon‐Based Optoelectronic Synapses for Dynamic Visual Recognition

**DOI:** 10.1002/advs.202414319

**Published:** 2025-01-22

**Authors:** Wenhao Liu, Jihong Wang, Jiahao Guo, Lin Wang, Zhen Gu, Huifeng Wang, Haiping Fang

**Affiliations:** ^1^ Haiping Fang School of Physics East China University of Science and Technology Shanghai 20023 China; ^2^ Key Laboratory of Smart Manufacturing in Energy Chemical Process Ministry of Education East China University of Science and Technology Shanghai 200237 China; ^3^ Zhejiang Lab Hangzhou 311100 China

**Keywords:** 2D heterostructure, C60, dynamic vision, graphene oxide, optoelectronic synapse

## Abstract

The human visual nervous system excels at recognizing and processing external stimuli, essential for various physiological functions. Biomimetic visual systems leverage biological synapse properties to improve memory encoding and perception. Optoelectronic devices mimicking these synapses can enhance wearable electronics, with layered heterojunction materials being ideal materials for optoelectronic synapses due to their tunable properties and biocompatibility. However, conventional synthesis methods are complex and environmentally harmful, leading to issues such as poor stability and low charge transfer efficiency. Therefore, it is imperative to develop a more efficient, convenient, and eco‐friendly method for preparing layered heterojunction materials. Here, a one‐step ultrasonic method is employed to mix fullerene (C60) with graphene oxide (GO), yielding a homogeneous layered heterojunction composite film via self‐assembly. The biomimetic optoelectronic synapse based on this film achieves 97.3% accuracy in dynamic visual recognition tasks and exhibits capabilities such as synaptic plasticity. Experiments utilizing X‐ray photoelectron spectroscopy (XPS), X‐ray diffraction spectroscopy (XRD), Fourier–transform infrared spectroscopy (FTIR), ultraviolet‐visible spectroscopy (UV‐vis), scanning electron microscopy (SEM), and transmission electron microscopy (TEM) confirms stable π‐π interactions between GO and C60, facilitating electron transfer and prolonging carrier recombination times. The novel approach leveraging high‐density π electron materials advances artificial intelligence and neuromorphic systems.

## Graphical abstract

This study introduces a one‐step ultrasonic method to synthesize a layered heterojunction composite film of fullerene (C60) and graphene oxide (GO). The resulting bionic optoelectronic synapse exhibits remarkable short‐term memory, long‐term learning, and synaptic plasticity, supported by extensive characterization techniques. This innovative approach enhances the application of π‐electron‐rich materials in artificial intelligence and neuromorphic systems.

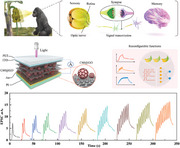

## 1 Introduction

The human nervous system is a sophisticated network of neurons and glial cells for receiving, processing, and transmitting information to regulate sensory organs. Among these, the visual sensory system (VSS) plays a critical role, as it accounts for ≈80% of external information acquisition through the eyes.^[^ ^1^, ^2^, ^3^ ^]^ In the human visual system, photoreceptor cells in the retina convert light stimuli into nerve impulses, which are then processed and stored in a highly parallel manner by multiple neurons via synapses.^[^ ^4^, ^5^, ^6^ ^]^ Biomimetic vision systems emulate the processes of the human eye and brain in processing visual information, thereby achieving functions analogous to those of biological vision.^[^ ^7^, ^8^ ^]^ Among them, synaptic devices, which are modulated by photon signals, simulate the retinal neurons in real eyes.^[^ ^9^, ^10^ ^]^ This type of artificial VSS possesses a range of synapse‐related functions, including paired‐pulse facilitation (PPF), short‐term plasticity (STP), long‐term plasticity (LTP), STP‐LTP conversion, excitatory postsynaptic current (EPSC), and other optical techniques. 2D materials, including MoS_2_,^[^ ^11^ ^]^ HfSe_2_,^[^ ^12^ ^]^ and graphene,^[^ ^13^ ^]^ can be coupled with different photoelectric molecules to form heterojunction layered materials. They have the potential to expand the scope of light detection by synapses, exhibit favorable memory plasticity, demonstrate flexibility with a low elastic modulus, and display superior control of photogenerated excitons and charge transport at the interface. It is anticipated that these materials will have a wide range of applications in the field of artificial optoelectronic synapses.^[^ ^14^, ^15^ ^]^ The majority of heterojunction materials are currently prepared through physical deposition,^[^ ^16^ ^]^ chemical vapor deposition,^[^ ^17^ ^]^ or a combination of these two processes.^[^ ^18^ ^]^ These methods are not only costly and rely on sophisticated equipment, but also frequently result in intrinsic defects such as poor grain boundary uniformity, poor stability, and low transfer efficiency, which collectively impede the large‐scale application of optoelectronic materials.^[^ ^16^, ^17^, ^18^, ^38^ ^]^ Therefore, it is necessary to develop an effective, rapid, and eco‐friendly method for obtaining heterojunction materials as the sensing layer of the optoelectronic synapse. Carbon‐based compounds, in particular graphene oxide (GO), are frequently employed as substrates for layered materials due to their malleable chemical structure, proclivity for non‐covalent bonding, tunable optoelectronic properties, and straightforward solution processes.^[^ ^19^ ^]^ It is a robust preparation scheme for heterojunction material layer based on GO and typical photoelectric materials. Notably, fullerene (C60) serves as an exemplary electron acceptor, making it an excellent candidate among typical photoelectric materials.

In this study, a homogeneous and flexible heterostructured photosensitive composite film was prepared using a one‐step ultrasonic method with C60 and GO. The optoelectronic device prepared based on this composite film exhibits short‐term memory, long‐term learning functions, and a high‐performance photo‐perception and synaptic plasticity system with a PPF index. Moreover, the experimental confusion matrix of C60@GO, comprising optoelectronic devices, exhibits a test accuracy of up to 97.3% for flashing letters in a video, thereby demonstrating its efficacy and practicality in dynamic visual recognition tasks. The analysis was conducted by X‐ray photoelectron spectroscopy (XPS), X‐ray diffraction spectroscopy (XRD), Fourier transform infrared spectroscopy (FTIR), ultraviolet‐visible spectroscopy (UV‐vis), scanning electron microscopy (SEM), and transmission electron microscopy (TEM). The findings reveal that C60 is stably non‐covalently bound to the GO sheet through π‐π interaction, electrons are transferred from GO to the C60 molecule, and the recombination time of photogenerated electrons and holes is prolonged. This study introduces an innovative technique and mechanistic research approach aimed at enhancing the application of π‐electron‐rich 2D materials within the optoelectronic domain. Consequently, it paves the way for novel opportunities and prospects in the fields of artificial intelligence and neuromorphism.

## 2 Results and Discussion

### 2.1 A Simulation of the Memory Process and the Function of the Synapses in Higher Organisms

**Figure 1:**
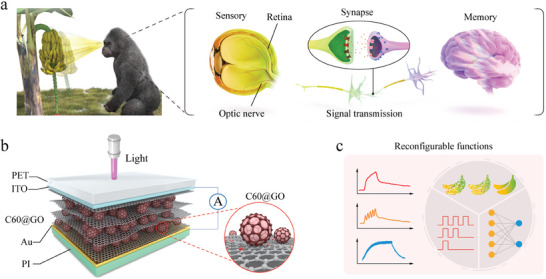
Schematic diagram of the C60@GO photoelectric device and its application in visual perception. a) Schematic diagram of the visual perception and memory system of higher organisms (gorillas). b) Structural diagram of the C60@GO photoelectric device and its partially enlarged internal view. c) Demonstration of the function of the C60@GO photoelectric device.

In the visual systems of higher organisms, the perception and transmission of information about the external environment is dependent upon the parallel processing of the retina, visual neurons, and various regions of the visual cortex in the brain. This mechanism enables the body to effectively construct the state of the surrounding world from visual perception, thereby facilitating the resolution of complex, unstructured problems in real‐life scenarios.^[^ ^20^, ^21^ ^]^ To elucidate this concept, consider the response elicited in a gorilla upon observing a cluster of oscillating bananas. As depicted in **Figure** **1**a, the movement of the bananas is detected and processed by the visual photoreceptors in the retina of a gorilla, subsequently transmitted to the brain via the optic nerve and synaptic connections. Within the brain, this information is relayed to the visual cortex, generating a visual stimulus. Distinct regions of the visual cortex are responsible for extracting relevant information pertinent to memory, learning, and recognition processes, including selective attention to specific stimuli, shape recognition, color differentiation, and motion perception. This processed information ultimately directs the motor responses of a gorilla accordingly.

To construct a flexible imitative visual memory system, we have assembled photoelectric devices with the requisite perception, memory, and visual processing functions (see Figure 1b). The input signal is selected as a representative violet light emitted by the laser and passed through the top PET and ITO layers into the device, and the output signal is collected by the top and bottom ITO and Au layers. The C60@GO composite film functions as the photoelectric detection layer of the optoelectronic synapse, thereby markedly enhancing the photoelectric properties. Furthermore, the preparation process is relatively convenient. Initially, the GO suspension prepared by the modified Hummers method was ultrasonically mixed with C60 aqueous dispersion to form a uniform C60@GO composite suspension.^[^ ^22^ ^]^ Following the self‐assembly and drying processes, a C60@GO composite film was produced. The stability of the C60@GO composite film is achieved by anchoring C60 to the GO plane via π‐π interactions. C60, a significant carbon‐based semiconductor, exhibits a wide spectral absorption band. GO flakes, which display graphene‐like aromatic sheets, possess multifunctional oxygen‐containing groups, including ─OH, ═O, and ─COOH. These groups enable GO to self‐assemble or co‐assemble with other materials, while also achieving spectral overlap absorption in the visible, ultraviolet, and near‐infrared (NIR) regions. An enlarged view of Figure 1b illustrates the microstructure of the C60 and GO composite film, wherein the C60 between the GO layers facilitates interfacial charge transfer, thereby enhancing the efficiency of the photoelectric effect. Considering that C60 possesses a lower lowest unoccupied molecular orbital (LUMO) compared to GO,^[^ ^23^, ^24^ ^]^ it functions as an electron acceptor, effectively trapping charge carriers. The combination with GO markedly prolongs the recombination time of electrons and holes, thereby enhancing the photoconductive effect and indicating potential applications in light‐induced synaptic behavior.

As illustrated in Figure 1c, the C60@GO device exhibits several artificial optoelectronic synaptic functions. The process by which a gorilla recognizes a banana and forms a memory in its brain can be simulated using the artificial synapses assembled by C60@GO. It can be employed as an optoelectronic synapse simulation cell and image‐processing logic cell. These functions are achieved through the exceptional optical response characteristics of the film and the coupling effect of the interface traps. The integration of these device units into an optoelectronic thin film array allows the construction of a hardware system with visual functions that fully exploit and optimize the device characteristics, thereby achieving multi‐functional biomimetic machine vision. This will assist in addressing the prospective challenges associated with high‐efficiency intelligent chips in terms of complexity and cost.

### 2.2 The Structure of the C60@GO Composite Film

**Figure 2:**
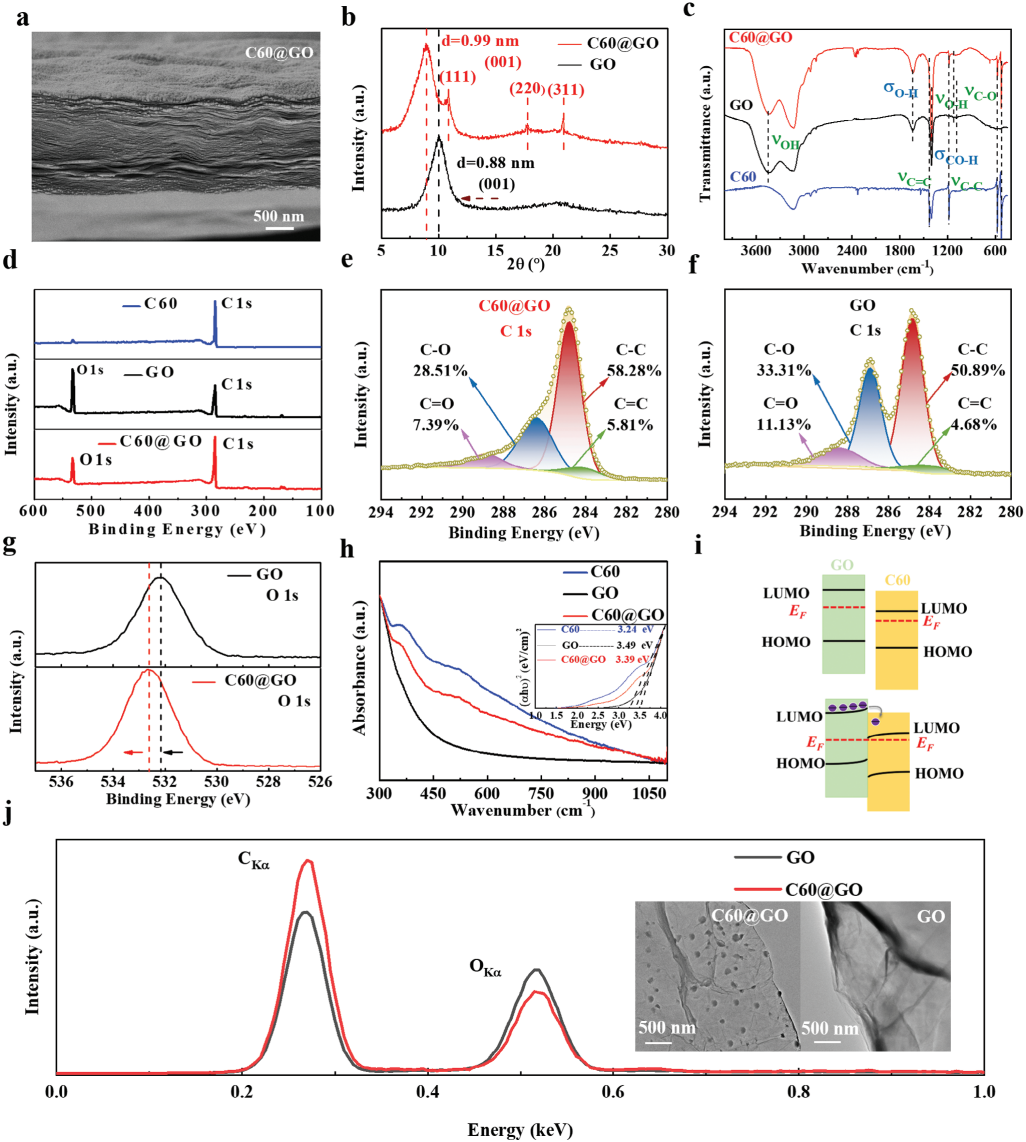
a) Cross‐sectional scanning electron microscopy (SEM) image of the graphene oxide (GO) film. b) X‐ray diffraction spectroscopy (XRD) spectra of the C60@GO composite film, with the GO film as the control. c) Fourier–transform infrared spectroscopy (FTIR) spectra of the C60@GO composite film, the GO film, and the C60. d) X‐ray photoelectron spectroscopy (XPS) spectra of the C60@GO composite film, the GO film, and C60. The C 1s XPS peak of e) the C60@GO composite film and f) the GO film. g) The O 1s and XPS peaks of the C60@GO composite film and the GO film. h) Ultraviolet‐visible spectroscopy (UV‐vis) of the C60 film, the GO film, and the C60@GO composite film. The illustration depicts the relationship between (*αhν*)^2^ and *hν*, which is employed to ascertain the band gap of the film. i) Energy arrangement diagram at the GO and C60 interfaces and schematic diagram surface transfer doping. EF is the Fermi level. j) Energy dispersive spectroscopy (EDS) spectral analysis on the layers of C60@GO and GO films; insets are transmission electron microscope (TEM) images of C60@GO (left) and GO (right).

The cross‐sectional morphology of the C60@GO composite film was analyzed by SEM, as illustrated in **Figure** **2**a. In comparison with the layered structure of the GO film (Figure S1, Supporting Information), the C60@GO image demonstrates a denser and more structured layered structure, which corresponds to the film structure of the photosensitive layer in Figure 1b. The film thickness is ≈1.5 µm, which suggests that C60 is adsorbed or embedded in the GO film in a uniform and orderly manner. In this system, the stable non‐covalent bonding in the C60@GO composite film is achieved by π‐π interactions, which fix the aromatic planes of C60 to GO. Furthermore, C60 exhibits a substantial electron‐withdrawing capacity,^[^ ^24^, ^25^ ^]^ which enables the attraction of electrons from the GO plane and the stabilization of GO dispersion. This ultimately results in the formation of a functionalized C60‐GO layer that is well‐suited for device fabrication. As illustrated in Figure 2b, the XRD analysis reveals structural alterations between the GO layers. In the GO, a distinct and broad diffraction peak is observed at 2*θ* = 9.9° (d‐spacing of 0.88 nm), which can be attributed to the (0 0 1) diffraction peak of GO. Following the introduction of C60 molecules, a reduction in the intensity of the primary peak was observed, accompanied by a notable sharpening and a slight shift in the peak position to a higher angle (2*θ* = 8.9°). This suggests an increase in the d spacing (0.99 nm), which may be attributed to the larger C60 molecules providing support between the GO layers. Furthermore, three distinct C60 diffraction peaks are discerned in the C60@GO film. The diffraction peaks at 2*θ* = 10.8, 17.7, and 20.8° correspond to the (111), (200), and (311) planes of C60, respectively.^[^ ^26^ ^]^ In Figure 2j, the TEM image illustrates that numerous small C60 particles are incorporated into the C60@GO composite film when compared to the GO film. It is noteworthy that the upper GO layer was elevated using adhesive tape for TEM analysis, which may have resulted in the displacement or removal of some C60 particles. Nevertheless, the overall distribution of C60 appears to be uniform across the GO layers. Additionally, the energy dispersive spectroscopy (EDS) analysis indicates that the carbon (C) content in the C60@GO film is markedly higher than that in the GO film, while the oxygen (O) content is significantly lower compared to the GO film. It provides evidence that C60 has been successfully embedded within the GO interlayer.

We also investigated the mixing of GO and C60 at the molecular level. As shown in Figure 2c, a broad absorption band at 3444 cm^−1^ in the FTIR spectra of GO is attributed to the hygroscopicity of GO caused by the adsorption of water molecules on the peripheral hydroxyl groups. The peak at 1638.43 cm^−1^ is related to the O─H bending vibration of the intercalated water molecules adsorbed by GO. Besides, the bending vibration absorption peak at 1398 cm^−1^ is related to the aliphatic CO─H bond, 1121 cm^−1^ is the stretching vibration peak of the O─H bond, and 1086 cm^−1^ is the C─O absorption peak.^[^ ^27^ ^]^ The intensities of all these absorption peaks in GO are relatively weak. In the FTIR transmission spectra of C60@GO, the intensities of these characteristic peaks are significantly enhanced. In addition, the characteristic absorption bands of the C60 molecule (≈525, 575, 1181, and 1427 cm^−1^) are clearly visible.^[^ ^28^ ^]^ These results further confirm the successful combination of C60 and GO.

To further evaluate the quality and chemical composition of the film, XPS was used to study the proportion of elements in C60@GO and the change in some binding energies. XPS full spectra in Figure 2d–g depicts that C60 is mainly composed of carbon, while C60@GO is mainly composed of carbon and oxygen. Compared with GO, the proportion of carbon and oxygen in C60@GO shows an opposite trend, with the proportion of carbon and oxygen increasing and decreasing respectively, which also confirms the robust combination of GO and C60. Moreover, a deconvolution analysis of the C 1s XPS spectra of the C60@GO composite film and the GO film was performed. The peak observed at 284.1 eV is likely associated with C═C bonds involving carbon atoms in sp^2^ hybridization, and the feature at 284.8 eV corresponds to C─C bonds with carbon atoms in sp^3^ hybridization. The peaks at 286.4 and 288.8 eV, represent C─O and C═O, respectively.^[^ ^29^, ^30^, ^31^ ^]^ These results provide important information about the changes in the chemical state of the film after the binding of GO and C60.

More interestingly, the strength of the C─C and C═C bonds in the C60@GO composite film exhibits a significant enhancement relative to that of GO. In contrast, the strength of the C─O bond diminishes from 33.31% to 28.51%, and the strength of the C═O bond decreases from 11.13% to 7.39%. It may be due to the adsorption or embedding of C60 in the GO film, which breaks the C─O and C═O bonds. In general, the decomposition of unstable oxygen functional groups leads to the restoration of the internal π‐conjugated network of GO, thereby enhancing the π‐π interaction between GO and C60. By comparing the O1s XPS spectra of the C60@GO composite film with that of GO, a significant positive shift can be observed in the binding energy. This positive shift indicates that electrons are transferred from GO to C60, which is related to the correlation between binding energy and electron density. The higher the electron density, the lower the binding energy. The doping process increases the electron density of C60 in the C60@GO composite and simultaneously reduces the electron density of GO. Since C60 has a significant electron adsorption capacity, this change in electron density promotes the spontaneous transfer of electrons from GO to C60.

The doping properties of GO have been studied by UV‐vis spectra. Figure 2h shows the transmission spectra of the GO film, the C60@GO composite film, and C60. They have relatively low absorption in the medium and long wavelength region, and the absorption peak of C60@GO is located between GO and C60. According to the formula: (*αhν*)^2^ = *hν−E*_g_, where *α* is the absorption coefficient, *hν* is the incident photon energy and *E*_g_ is the band gap.^[^ ^32^ ^]^ Extrapolating the line segment of the (*αhν*)^2^ graph to the *hν* axis gives the x‐intercept corresponding to *E*_g_, as shown in Figure 2h. According to the calculation results, the band gaps of C60, C60@GO, and GO are 3.34, 3.39, and 3.49 eV, respectively. It also proves that C60 and GO are successfully combined and effectively improve the band gap of GO. The Fermi level, typically represented by the symbol *E*_F_ exerts control over the conductivity of materials. In our system, the difference in Fermi level between C60 and GO is a significant factor influencing charge transfer. Figure 2i illustrates the anticipated charge transfer at the GO@C60 interface. Given the LUMO energy level of C60, electrons in the GO donor state are extracted by the organic molecules. Upon contact, it is anticipated that the energy level difference will facilitate electron transfer from the donor state of GO to the LUMO‐related acceptor state of C60. It will influence the relative alignment of the Fermi levels, leading to the formation of a space charge region (SCR) at the interface, accompanied by charge accumulation and band bending.

### 2.3 The Transition from Short‐Term Memory (STM) to Long‐Term Memory (LTM) is Simulated in the Optoelectronic Synapse Device

**Figure 3:**
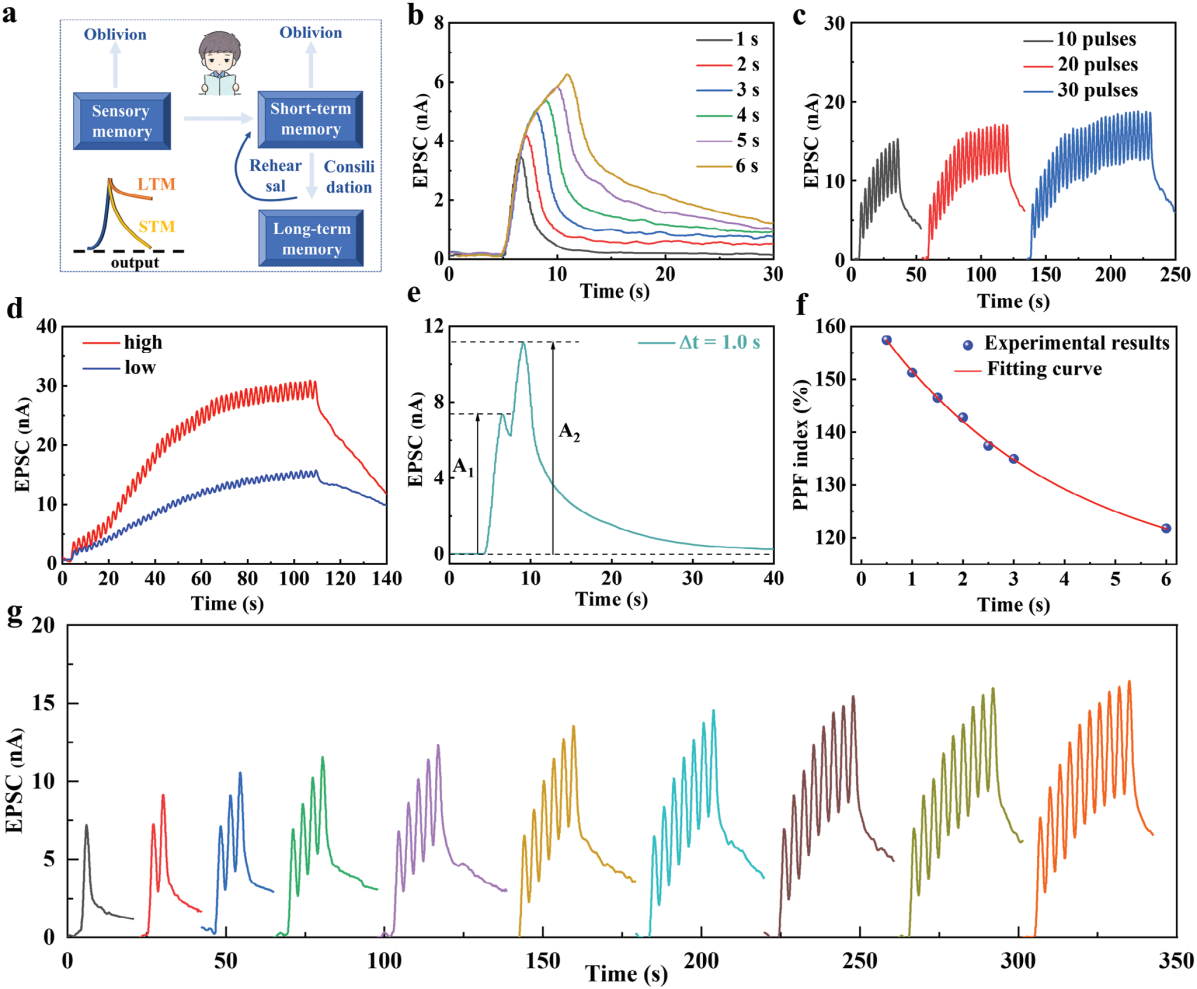
a) Schematic representation of the memory pattern in the human brain. b) Visible light duration‐related plasticity (5 mW cm^−2^). c) Current response of low‐frequency and high‐frequency input light signals representing memory overdrive (8 mW cm^−2^). d) High‐intensity stimulation (10 mW cm^−2^) and low‐intensity stimulation (2 mW cm^−2^) related plasticity. e) Excitatory postsynaptic current (EPSC) induced by two successive laser spikes with a spike time interval (Δ*t*) of 1.0 s. f) Variation of the paired‐pulse facilitation (PPF) index with the interval time between two pulse spikes. g) Visible light irradiation time‐related plasticity (8 mW cm^−2^). b‐g are carried out at a bias voltage of 0.1 V, under a 395 nm.

In the process of human learning and memory, visual information that is immediately acquired and temporarily stored in the hippocampus will gradually disappear unless the information is constantly repeated over a period of time. These memories are converted into long‐term memories (LTM) through rehearsal learning (**Figure** **3**a),^[^ ^33^, ^34^ ^]^ which allows the system to recall past experiences and thus achieve multi‐level dynamic recognition. Therefore, the photoreceptive and synaptic properties of the C60@GO device were investigated by applying a fixed bias voltage of 0.1 V to track the changes in postsynaptic current, simulating biological visual functions. To fully describe the optoelectronic behavior of the device, we used monochromatic light of different wavelengths (395, 532, and 635 nm, with an optical intensity of 30 mW cm^−2^). As can be seen in Figure S2a (Supporting Information), the C60@GO device exhibits different EPSC values when excited by different wavelengths at the same optical power intensity, with the highest response observed at 395 nm. It has frequency‐dependent plasticity to visible light and can convert optical stimuli into neural impulses, thereby extending the visual perception range of artificial synapses. The postsynaptic currents of C60@GO devices were stimulated by ultraviolet light with a pulse width of 2 s at different light intensities (Figure S2b, Supporting Information). When the optical signal power was increased from 20 to 80 mWcm^−2^, the EPSC continued to increase, possibly because the photosensitive part of the C60@GO device produces more photogenerated electron‐hole pairs, thereby increasing the number of carriers trapped in the device. Upon stimulation with relatively weak ultraviolet (UV) light, the C60@GO device produces a postsynaptic current that rapidly returns to its baseline conductance state, indicative of short‐term memory (STM) functionality. In contrast, exposure to high‐intensity UV light potentiates synaptic connections, characterized by amplified postsynaptic currents and prolonged relaxation times, reflecting LTM capabilities. It suggests that more photogenerated carriers are generated at high light intensities, and prolonged exposure to UV light further enhances the amplitude of excitatory postsynaptic currents (EPSCs) and extends decay times (Figure 3b). By continuously turning the UV light on and off, more presynaptic light pulses can be induced. To verify the relationship between STM and LTM, different numbers of UV light pulses to the C60@GO device were applied, 10, 20, and 30 times (Figure 3c). It indicates that the EPSC of the device continues to increase, the conductivity is significantly enhanced, and the decay time of the device current is also significantly prolonged. By increasing the number of light pulses, the memory retention time of the device is improved by two orders of magnitude. The transition from STM to LTM is not only related to the number of times the device learns but also to the strength of the learning. Figure 3d depicts the dependence of the memory behavior of the device on the strength of the learning. Two different intensities of pulse stimuli were employed, each administered 50 times. It can be seen that the EPSC value under high‐intensity pulse stimulation (10 mW cm^−2^) is always greater than that under low‐intensity pulse stimulation (2 mW cm^−2^), but the response values of both are consistent with the trend of LTM, which is always increasing. Under high‐intensity light stimulation, a significant number of electrons in the C60@GO material are excited, leading to the generation of a high density of electron‐hole pairs. This process facilitates rapid carrier migration and results in a substantial photocurrent. Concurrently, there are pronounced alterations in the internal charge distribution and electric field within the optoelectronic synapse. The swift accumulation of charge over a short duration induces a marked increase in the intensity of the internal electric field. Upon the cessation of the high‐intensity stimulus, the abrupt shift in the internal electric field prompts rapid recombination of electrons and holes, accompanied by accelerated diffusion and recombination of carriers, which ultimately causes a rapid decline in the photocurrent. In contrast, under low‐intensity light stimulation, the limited generation of carriers results in minimal changes to the internal electric field, and the recombination rate of carriers is comparatively slow, leading to a more gradual decay of the current. Besides, the duration of memory induced by high‐intensity pulse stimulation is significantly longer than that induced by low‐intensity pulse stimulation, indicating that the memory function can be regulated by changing the intensity of the input optical signal. These results strongly suggest the potential application of this device in advanced artificial synapses and its function of enhancing memory capacity.

PPF is one of the typical functions of STP in biological nervous systems, reflecting the ability to process temporal information between two consecutive stimuli. Two consecutive light pulses were applied to the C60@GO device, and a specific time interval was allowed for monitoring of the photocurrent response (Figure 3e). In the EPSC of two consecutive weak light pulses (30 mW cm^−2^, Δ*t* = 1 s), the current peak of the second pulse (A_2_) is higher than that of the first pulse (A_1_). It may be attributed to an insufficient interval time, which leads to carrier accumulation, accompanied by an increase in carrier recombination time. The ratio of A_2_/A_1_ is referred to as PPF,^[^ ^35^ ^]^ which is typically associated with alterations in the stimulus interval and is crucial for the temporal decoding of visual signals.^[^ ^36^, ^37^ ^]^ By testing the time‐varying EPSC curve and extracting the PPF index for different intervals, it was found that the PPF index of the C60@GO device was as high as 157%, which exceeds that of many newly reported optoelectronic synapses (Figure 3f).^[^ ^38^, ^39^, ^40^ ^]^ These findings demonstrate the considerable potential of the C60@GO device for applications in optically induced synaptic behavior.

To further investigate the synaptic‐dependent changes, the number of light pulses was increased from 1 to 10. As the number of light pulses increased, the photocurrent in response to the device exhibited a continuous rise, with the incremental change in current per light pulse remaining consistent, attributable to the unvarying light intensity delivered by each pulse. When subsequent pulses were applied before the previous pulse had finished, the accumulation of current resulted in a prolonged memory time for the device. It indicates that the C60@GO device is transitioning from STP to LTP (Figure 3g). A series of presynaptic pulses applied to the C60@GO device increased postsynaptic current, thus strengthening the synaptic connection (i.e., ‘learning’). When the optical stimulation was removed, the postsynaptic current gradually decreased (i.e., ‘forgetting’), indicating the presence of an STM process. After the ‘forgetting’ process, the same presynaptic stimulation was applied and the postsynaptic current quickly recovered and exceeded the previous memory level (i.e., ‘relearning’), demonstrating improved learning and memory abilities. Meanwhile, a slowing of the decay rate of the postsynaptic current could be observed after the removal of the light stimulus, marking the onset of the LTM process. Similar to human learning and memory processes, the C60@GO device can effectively promote learning and memory strength through repeated ‘learn‐forget‐relearn’ processes that are influenced by learning time and learning interval. Following several relearning phases, the relaxation time curve during the forgetting process exhibited a gradual increase, suggesting a transition from STM to LTM within the C60@GO device. Moreover, a forgetting process analogous to that of the brain was observed during the current decay process. Although forgetting is often thought of as a defect in memory, research indicates that it is essential for normal brain function. Forgetting, like learning, is part of the memory system in the brain. In Figure 3d–f, the current decay rate gradually slows down over time, which is consistent with the forgetting law of ‘fast first, slow later’ during the forgetting process. The transition from STP to LTP occurs during changes in peak power, duration, and stimulus frequency. Therefore, by adjusting the intensity, duration, and number of input light pulses, the synaptic weight can be modified, thereby enabling the achievement of learning and memory functions akin to those of biological neural systems. Moreover, the C60@GO device exhibits remarkable durability, stability, and reproducibility, sustaining optimal performance even when subjected to bending stresses (refer to Figures S4–S7, Supporting Information). Specifically, this heterosynapse can be used as an artificial neuron to realize a neuromorphic network with visual learning and recognition capabilities. Due to its high PPF characteristics, it is expected to achieve efficient decoding of temporal information.

### 2.4 Memory‐Dependent Dynamic Vision Recognition

**Figure 4:**
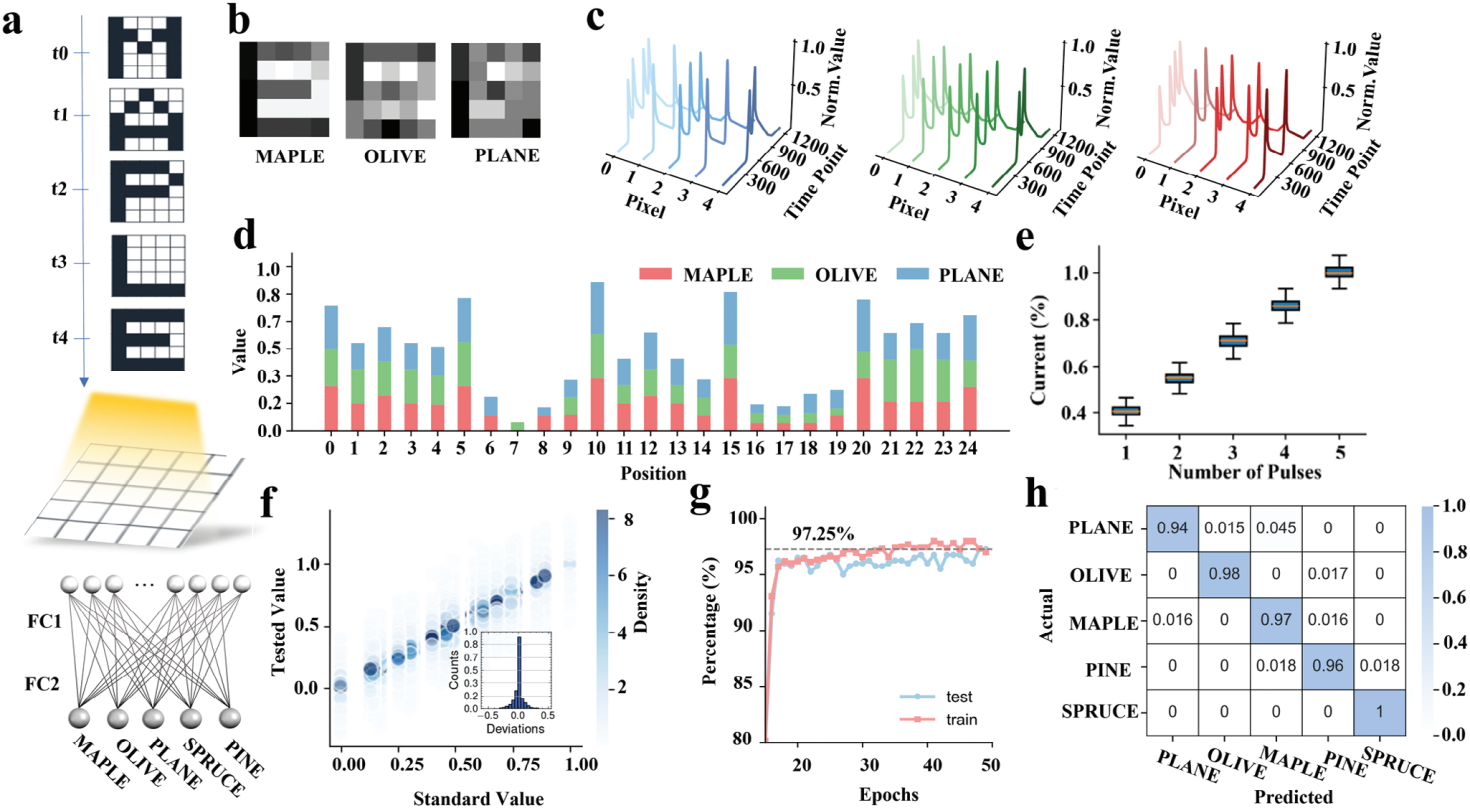
a) Videos playing ‘MAPLE’, ‘OLIVE’, ‘PLANE’, ‘PINE’, and ‘SPRUCE’ letter‐by‐letter, all ending with ‘E’, are used as input to the C60@GO array. b) Only the heterosynaptic current intensity distribution of the final frame, recorded after the display of the letter ‘E’, is used as input for recognition by the readout network. Due to space limitations, only the results for ‘MAPLE’, ‘OLIVE’, and ‘PLANE’ are shown. c) Output currents of the first 5 pixels in the C60@GO array recorded while playing the words ‘MAPLE’, ‘OLIVE’, and ‘PLANE’ letter‐by‐letter. The light pulses illuminate the C60@GO for 100 ms, the frame‐to‐frame rate is 10 Hz, and the bias voltage is 0.1 V. d) Feature vectors of the last frame (letter ‘E’) for ‘MAPLE’, ‘OLIVE’, and ‘PLANE’. The vectors are obtained by normalizing the heterosynaptic currents that are recorded after the 5th frame in c). e) Box plot illustrating the variation in output current of the last frame as a function of the number of optical pulses received previously. f) The dataset deviation was recorded by repeating each experiment 100 times under bias voltage Vbias = 0.1 V, for five optical pulses with a duration of 100 ms and a repetition rate of 10 Hz. g) Training and test accuracy for datasets recorded in the same video classification task. h) Confusion matrix in the experiment.

Given the heterosynaptic's ability to record single‐point temporal information, it can be inferred that an array of these heterosynaptic units could exhibit a 2D temporal accumulation effect. To explore and validate this hypothesis, we conducted a dynamic vision recognition experiment designed to demonstrate this phenomenon. In this experiment, we utilized videos that sequentially displayed words letter‐by‐letter as input. The words used in the videos were ‘PLANE,’ ‘OLIVE,’ ‘MAPLE,’ ‘PINE,’ and ‘SPRUCE’–all tree names ending with the letter ‘E’, displayed with a resolution of 5 × 5 pixels. These videos projected light onto our C60@GO array, generating spatiotemporal current signals, which were then processed by a simple readout network to classify the types of trees (**Figure** **4**a). Unlike traditional image sensors, where the final frame typically only captures the information of the letter ‘E,’ losing prior context, our C‐G system retains information about all preceding letters due to its inherent dynamic memory (Figure 4b). For instance, when the video displays ‘M‐A‐P‐L‐E,’ the classification vectors for the final frame are influenced by the entire sequence of letters rather than just the last ‘E’ (Figure 4c). Consequently, each video encodes unique spatiotemporal information. This distinctive behavior arises because the output currents recorded during the display of the final letter “E” are affected not only by the current optical input but also by the sequence and timing of all previous inputs, resulting in unique current maps for each video.

This is demonstrated in Figure 4d. The feature vectors of the last frame (letter ‘E’) for ‘MAPLE,’ ‘PLANE,’ and ‘OLIVE’ are presented in Figure S9 (Supporting Information). In this figure, the *x*‐axis represents the pixel position index in the last frame, while the length of the color bar indicates the current intensity corresponding to each word at that position. The visualization effectively highlights the distinct current distributions among the words across different pixel positions, providing a clear and intuitive comparison. Feature vectors for ‘PINE,’ and ‘SPRUCE’ are also shown in supplementary material (Figure S9, Supporting Information). This device not only shows clear decision boundaries between categories but also exhibits strong linearity in the mapping between pulse and current spaces. Linearity ensures optimal variance updates, speeding up convergence and reducing overfitting,^[^ ^41^, ^42^, ^43^, ^44^ ^]^ while also enhancing the model's generalization ability.^[^ ^45^ ^]^ As shown in Figure 4e, this figure presents the current values of the last frame captured after applying 1 to 5 pulses, across multiple experimental trials. The Pearson Correlation Coefficient of the data in the figure was calculated to be 0.9472, indicating a high degree of linear correlation close to 1. This high linearity suggests that the increase or decrease in pulse number leads to a monotonous change in the current, which ensures a regular mapping between the pulse space and current space, contributing to the model's generalization capability and improving its performance on unseen data.

A convolutional neural network (CNN) is employed to exploit spatio‐temporal variations in dynamic vision recognition. The use of CNNs brings us closer to mimicking advanced biological visual systems, especially the human visual system.^[^ ^46^, ^47^, ^48^, ^49^ ^]^ First, this visual system aids the brain in recognizing “what” information, such as the shape, color, and details of objects. This is analogous to the feature extraction phase in CNNs, where convolutional layers identify local features in an image, such as edges, corners, and textures.^[^ ^50^ ^]^ Second, in this system, information is processed sequentially through different cortical regions, with each region responsible for extracting features at different levels, ultimately leading to object recognition. This is similar to the multi‐layer convolutional structure of CNNs, where different levels of features are extracted through successive layers, and the final output is processed through fully connected layers for classification or regression tasks.^[^ ^51^ ^]^ Upon capturing time‐sensitive current distributions through a device array, the extracted feature maps of the current are fed into the CNN. The network then performs feature extraction and hierarchical processing, ultimately generating the recognition results. To train this CNN, a custom dataset was constructed. Specifically, for each word – ‘PLANE’, ‘OLIVE’, ‘MAPLE’, ‘PINE’, and ‘SPRUCE’ – the same video sequence was shown 100 times, and the current maps of the final letter ‘E’ were recorded 1.5 s after the last letter pulse. In each video, the pulse and rest durations for each letter were 1.5 s, respectively. This dataset incorporates noise and jitter from the light source, our C60@GO array, and the current meter, making the training results more robust to real‐world environmental variations. This is demonstrated in Figure 4d, where the *x*‐axis represents the normalized current values of each pixel in the last frame, and the y‐axis represents the normalized test current values after multiple repetitions. The standard deviation of the difference between the test values and the standard values is 0.08, indicating that the samples are minimally affected by experimental noise. This consistency further reduces the sensitivity to noise and lowers the overfitting risk in the mode training process. Based on the constructed dataset, the readout CNN was trained to discriminate between the videos. The ANN comprised 25 input nodes corresponding to the 25 heterosynaptic output currents of the C‐G system and 5 output nodes for video classification. The CNN network consisted of a single convolutional layer with 15 channels and 2 × 2 kernels. After flattening, the fully connected section includes one layer with 123 features, followed by a decision layer. After 50 training epochs, the recognition accuracy reached 97.3%, as shown in Figure 4g. The experimental confusion matrices indicate strong test accuracies, demonstrating the system's effectiveness in dynamic vision recognition tasks (Figure 4h).

## 3 Conclusion 

In summary, we employed a one‐step ultrasonic method in conjunction with self‐assembly to fabricate flexible films of C60@GO photoelectric layered heterojunctions, which is convenient and environmentally friendly. The optoelectronic synapse assembled on this film exhibits excellent STM, LTM ability, and synaptic plasticity. Besides, the experimental confusion matrix of the C60@GO device shows a high accuracy of 97.3% in video analysis tasks, demonstrating the efficiency of its dynamic visual recognition. Due to its high PPF function, it is expected to achieve efficient time information decoding. Moreover, XPS, XRD, FTIR, UV‐vis, and SEM analyses reveal that GO and C60 are robustly bonded through π‐π interactions, and electrons are transferred from GO to C60, thereby prolonging the recombination time of carriers and holes. This study reveals that this method can significantly enhance the application scope of 2D π‐electron‐rich materials within the field of optoelectronics.

We expect that the plasticity and functionality of the optoelectronic synapses can be further enhanced by optimizing the heterojunction structure of the materials or by selecting carbon‐based materials with better performance. Given the inherent simplicity of this method, its combination with other established techniques is anticipated to significantly improve the responsiveness and synapse function of optoelectronic devices. These findings therefore provide a straightforward way to optimize the optoelectronic properties of materials and open up new ideas and application prospects for further research in the fields of artificial intelligence and neuromorphism.

## 4 Experimental Section

### Materials

The graphene oxide (GO) suspension (5 mg mL^−1^) was prepared by an improved Hummers method as described in the previous research.^[^ ^52^, ^53^, ^54^ ^]^ Ultrapure water with a remarkable electrical resistivity of 18.2 MΩ cm^−1^ was obtained from a MERCK ultrapure water system. Fullerene (C60) was supplied by Aladdin Biological Technology Co., Ltd. (Shanghai, China). All materials were used without additional purification.

### Preparation of the C60@GO Composite Film and the C60@GO Optoelectronic Synapse

Preparation of the C60@GO composite film. The C60@GO composite film was prepared through a straightforward process. The initial stage of the process involved the preparation of two separate solutions: C60 aqueous dispersion (1 mm) and GO suspension (5 mg mL^−1^). Each solution was sonicated at room temperature for 30 min with a power setting of 60 W. Thereafter, the two solutions were combined in a 1:1 volume ratio and subjected to an additional 30 min of sonication under the same conditions. Afterward, an equal volume of ultrapure water was added to the mixture, which was then subjected to another 30 min of sonication to achieve a homogeneous C60@GO suspension. Subsequently, the suspension was divided into equal‐volume droplets and transferred onto release paper using the drop‐casting technique, thereby allowing for self‐assembly before drying for 12 h, which resulted in the formation of the C60@GO composite film. In contrast, the GO film was prepared using the same procedure without the addition of C60, serving as a control group.

### Preparation of the C60@GO Optoelectronic Synapse

The flexible ITO‐coated PET substrate was subjected to a sequential cleaning process involving acetone and isopropyl alcohol for 20 min each. This process was conducted by the standard protocol for cleaning such materials. Subsequently, the material was rinsed with ultrapure water and dried using nitrogen gas. The C60@GO optoelectronic synapse was constructed with a PET/ITO/C60@GO/Au/PI structure (Figure 1b), wherein the C60@GO composite film functioned as the photoelectric layer. The aforementioned layer, which has a dimension of 1 × 1 cm^2^, was held in place by the upper and lower layers of PET and PI. The C60@GO optoelectronic synapse array was packaged in the same way as the individual devices, forming 5 × 5 square arrays on a larger plane.

### Characterization of Films

Scanning electron microscopy (SEM) was employed to obtain images of the C60@GO composite film and the GO film. The SEM used was a ZEISS Gemini 300, Germany, with an accelerating voltage of 3 kV. The morphological details of the C60@GO and GO films were observed via FEI Talos F200S G2 (FEI Co., Ltd., USA) transmission electron microscope (TEM). The elemental analysis was performed by using the TEM coupled with an energy dispersive spectroscopy (EDS). The FTIR spectra of the GO film, the C60@GO composite film, and C60 were obtained from a Nicolet is50 spectrophotometer (Thermo Scientific, USA). X‐ray diffraction patterns were obtained for both the C60@GO composite film and the GO film within the 2*θ* range of 5 to 30° using an X‐ray diffraction instrument (XRD, Rigaku Ultimate IV, Japan) with a Cu Kα radiation source (λ = 0.15418 nm). An X‐ray photoelectron spectroscopy (XPS) spectrometer (Thermo Fisher ESCALAB 250Xi, USA) was employed for surface analysis of the C60@GO composite film and the GO film. The ultraviolet‐visible (UV‐Vis) absorbance spectra were recorded in the 300–1100 nm range for the GO film, the C60@GO composite film, and the C60 (UH5300, Hitachi, Japan).

### Measurement of Properties of the C60@GO Optoelectronic Synapse

The photocurrent versus time curves of the C60@GO optoelectronic synapse were recorded at varying optical powers and irradiation times using a Keithley 6517B light meter (Tektronix, USA). In order to minimize external interference due to poor signal transmission, a gold probe was used for the measurement of photocurrent. The incident light was generated by 395, 532, and 635 nm lasers with adjustable power densities, carefully calibrated using photodiode power sensors (Thorlabs, S120VC, and PM100D), and the illumination position and area were held constant for all measurements. It should be noted that the measurement environment was illuminated exclusively by the laser. Furthermore, the optoelectronic synapse was subjected to testing under controlled conditions of room temperature (25 °C), 20% humidity, and atmospheric pressure.

### Dataset Construction

The input was provided in the form of videos that displayed the words sequentially, with each letter displayed individually. The vocabulary employed in the videos comprised the following five tree names, all of which end in the letter ‘E’: ‘PLANE,’ ‘OLIVE,’ ‘MAPLE,’ ‘PINE,’ and ‘SPRUCE’. These were displayed with a resolution of 5 × 5 pixels.

## Conflict of Interest Statement

The authors declare no conflict of interest.

## Author Contributions

W.L. and J.W. contributed equally to this work. W.L. wrote, reviewed, and edited the manuscript, wrote the original draft, and performed visualization, validation, methodology, investigation, and formal analysis; J.W. wrote, reviewed, and edited the manuscript, wrote the original draft, and performed visualization, validation, methodology, investigation, formal analysis, and conceptualization; J.G. performed methodology and investigation; L.W. wrote, reviewed and edited the manuscript, performed software, resources, methodology, formal analysis, and conceptualization; Z.G. wrote, reviewed and edited the manuscript, performed supervision, software, resources, project administration, funding acquisition, formal analysis, and conceptualization; H.W. performed supervision, resources, project administration, methodology, funding acquisition, formal analysis, and conceptualization; H.F. performed supervision, resources, project administration, methodology, funding acquisition, formal analysis, and conceptualization.

## Supporting information



Supporting Information

## Data Availability

The data that support the findings of this study are available from the corresponding author upon reasonable request.
