# Supplementary material for: Efficient Carbon‐Based Optoelectronic Synapses for Dynamic Visual Recognition
Source: Adv Sci (Weinh). 2025 Jan 22;12(11):2414319. doi: 10.1002/advs.202414319 (PMC11923932; doi:10.1002/advs.202414319)
Supplement: Supplementary file 1 — Supporting Information [file ADVS-12-2414319-s001.docx]

Supporting Information

**Efficient Carbon-Based Optoelectronic Synapses for Dynamic Visual Recognition**

Wenhao Liu^†^, Jihong Wang^†*^, Jiahao Guo, Lin Wang^*^, Zhen Gu^*^, Huifeng Wang, and Haiping Fang


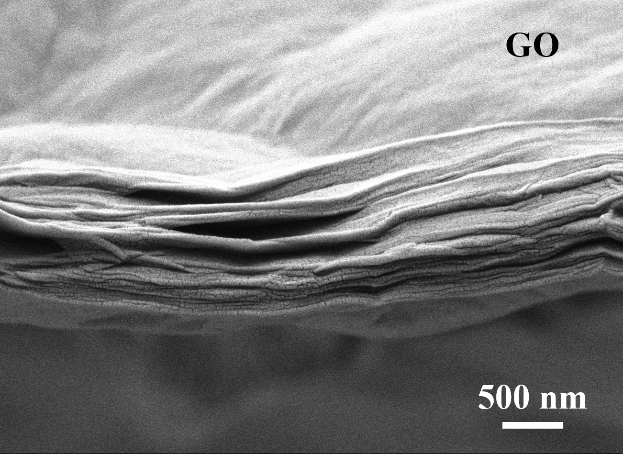


**Figure S1.**Cross-sectional SEM image of GO film.


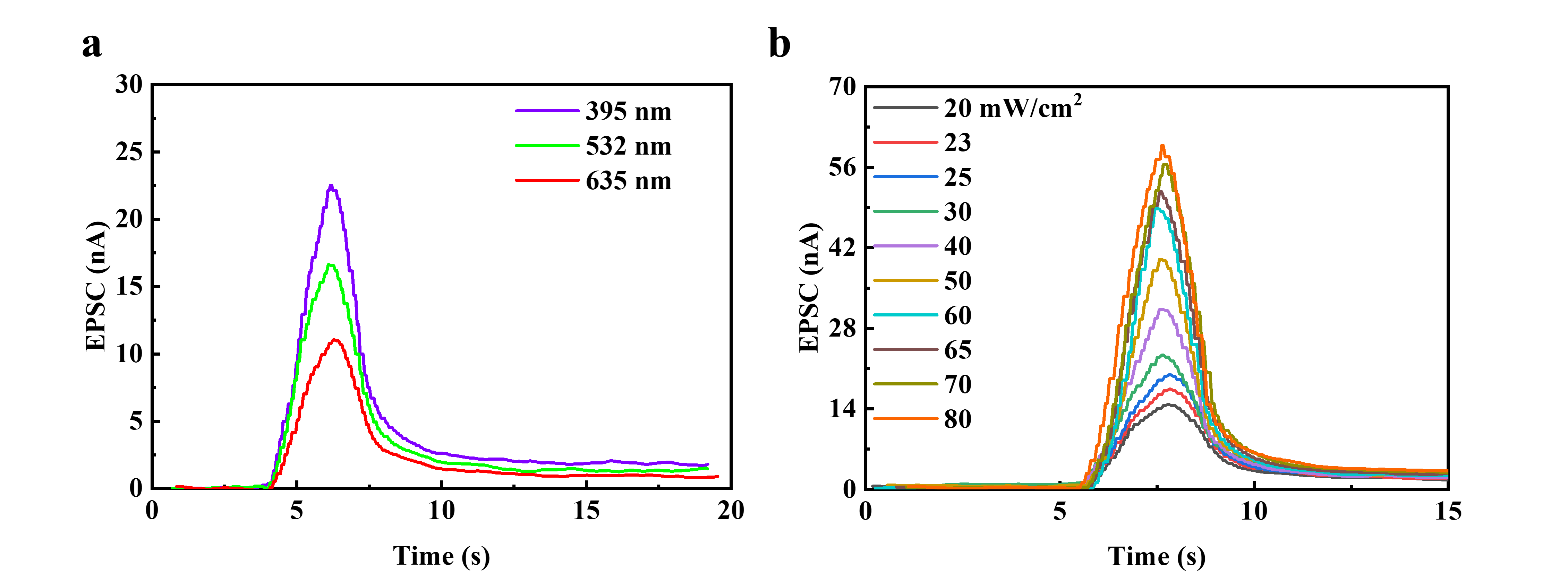


**Figure S2.** The EPSC of the C60@GO optoelectronic synapse exhibits a change in response to changes in laser (a) Visible light frequency-related plasticity (b) Visible light intensity-related plasticity.


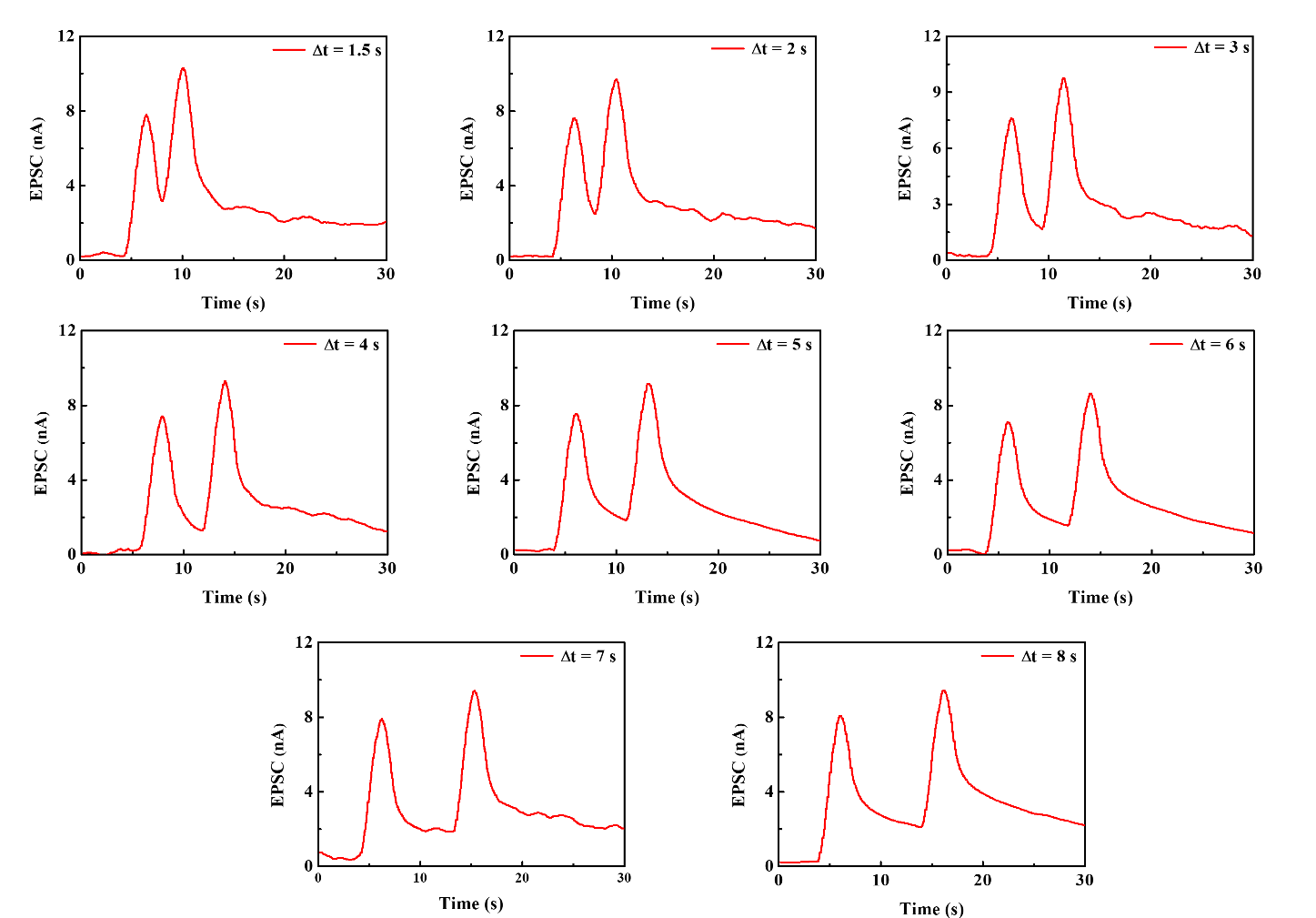


**Figure S3.** EPSC induced by two successive laser spikes (395 nm, 10 mW cm^-2^) with different spike time interval (Δ*t*).

**Figure S4.** The EPSC of the C60@GO optoelectronic synapse under various bending angles under 395 nm illumination (optical pulse of 1 s, 5 mW cm^-2^).


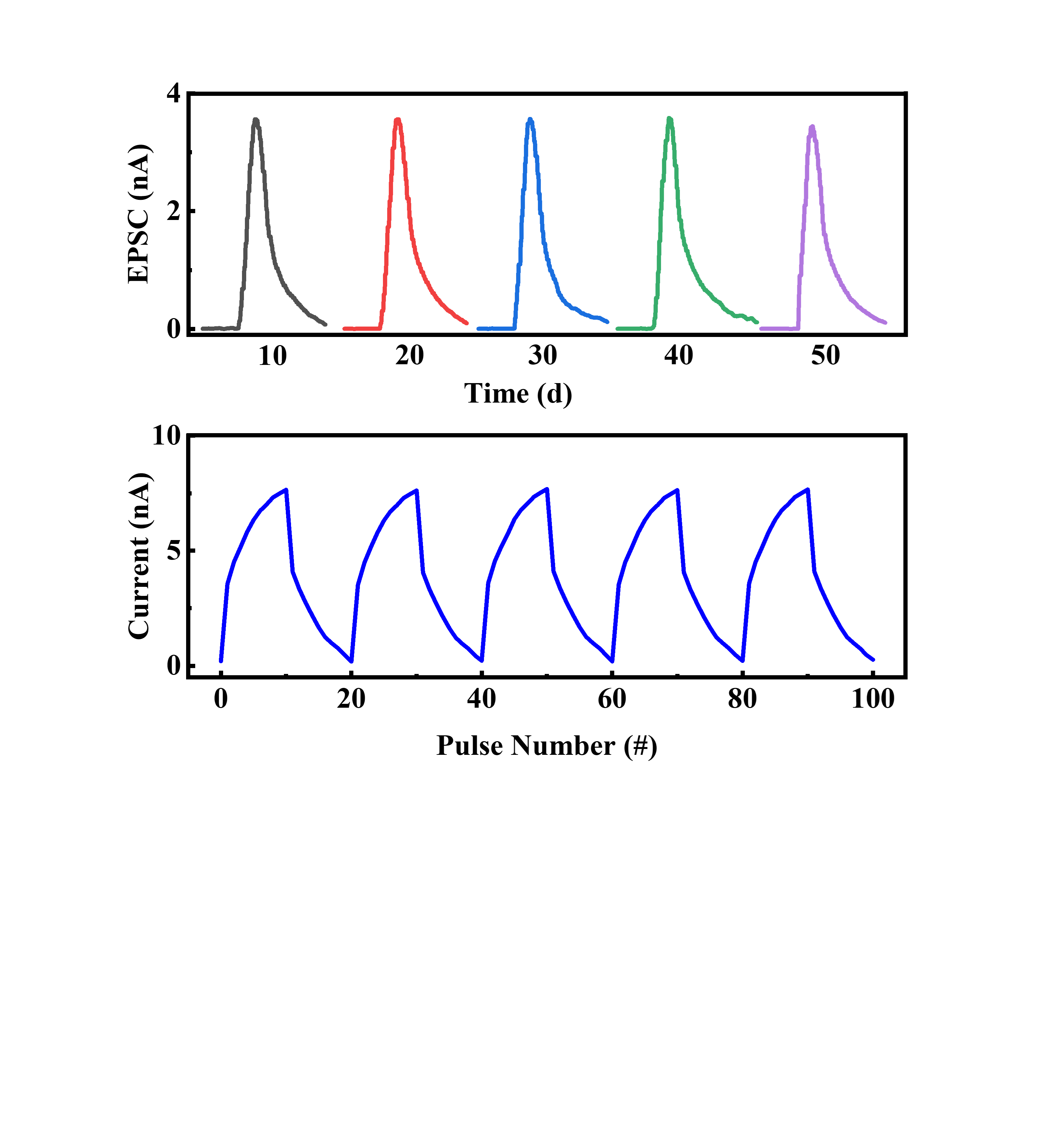


**Figure S5.** Consecutive 5 cycles of UV-induced potentiation (395 nm, duration: 1 s, 0.5 Hz) and negative voltage-induced depression (-0.05 V, duration: 0.5 s, 0.5 Hz) in C60@GO optoelectronic synapse demonstrating exceptional repeatability, reliability, and cycle uniformity.
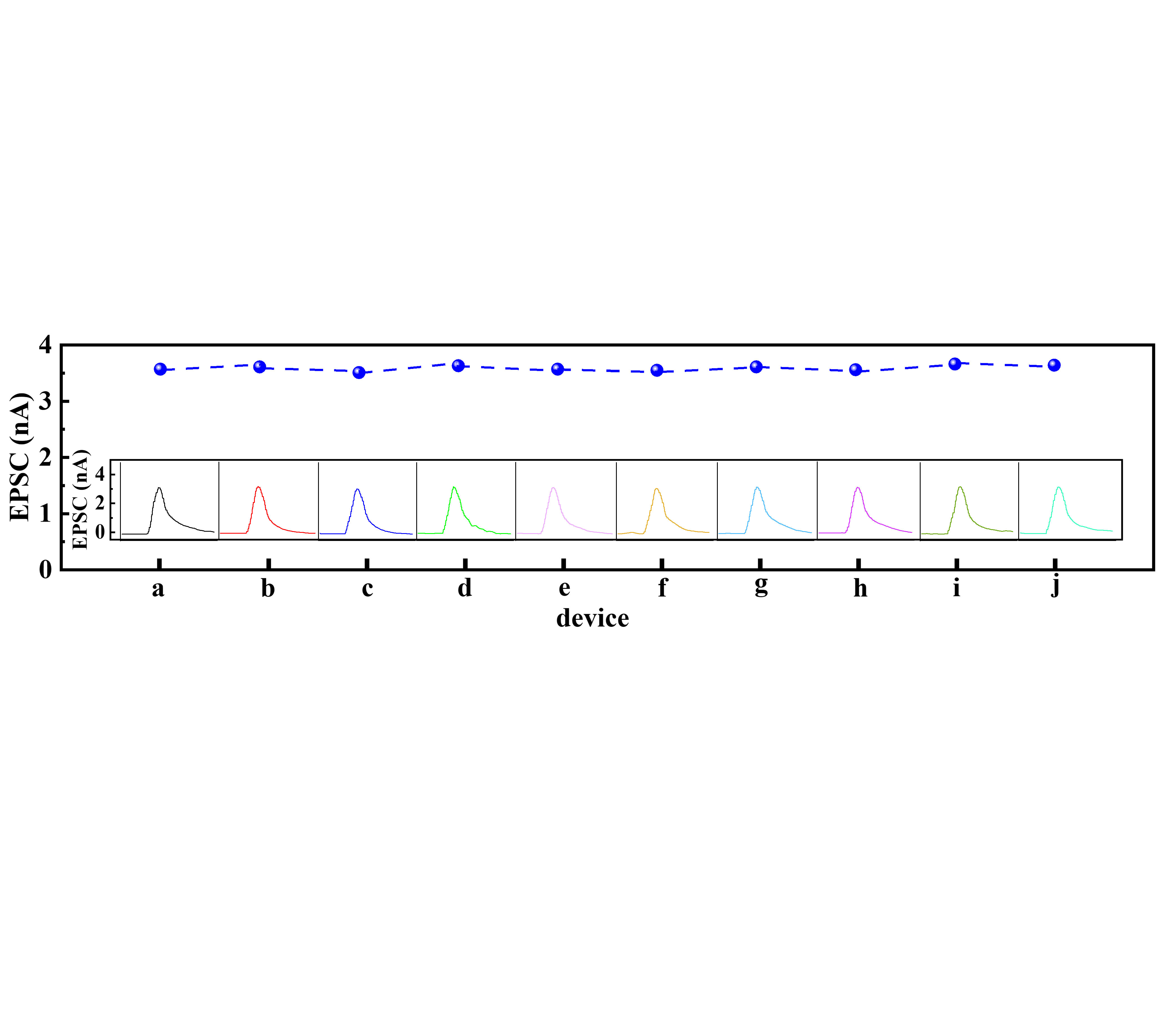


**Figure S6.** EPSC of 10 randomly chosen C60@GO optoelectronic synapses, demonstrating good device-to-device uniformity under 395 nm illumination (optical pulse of 1 s, 5 mW cm^-2^).


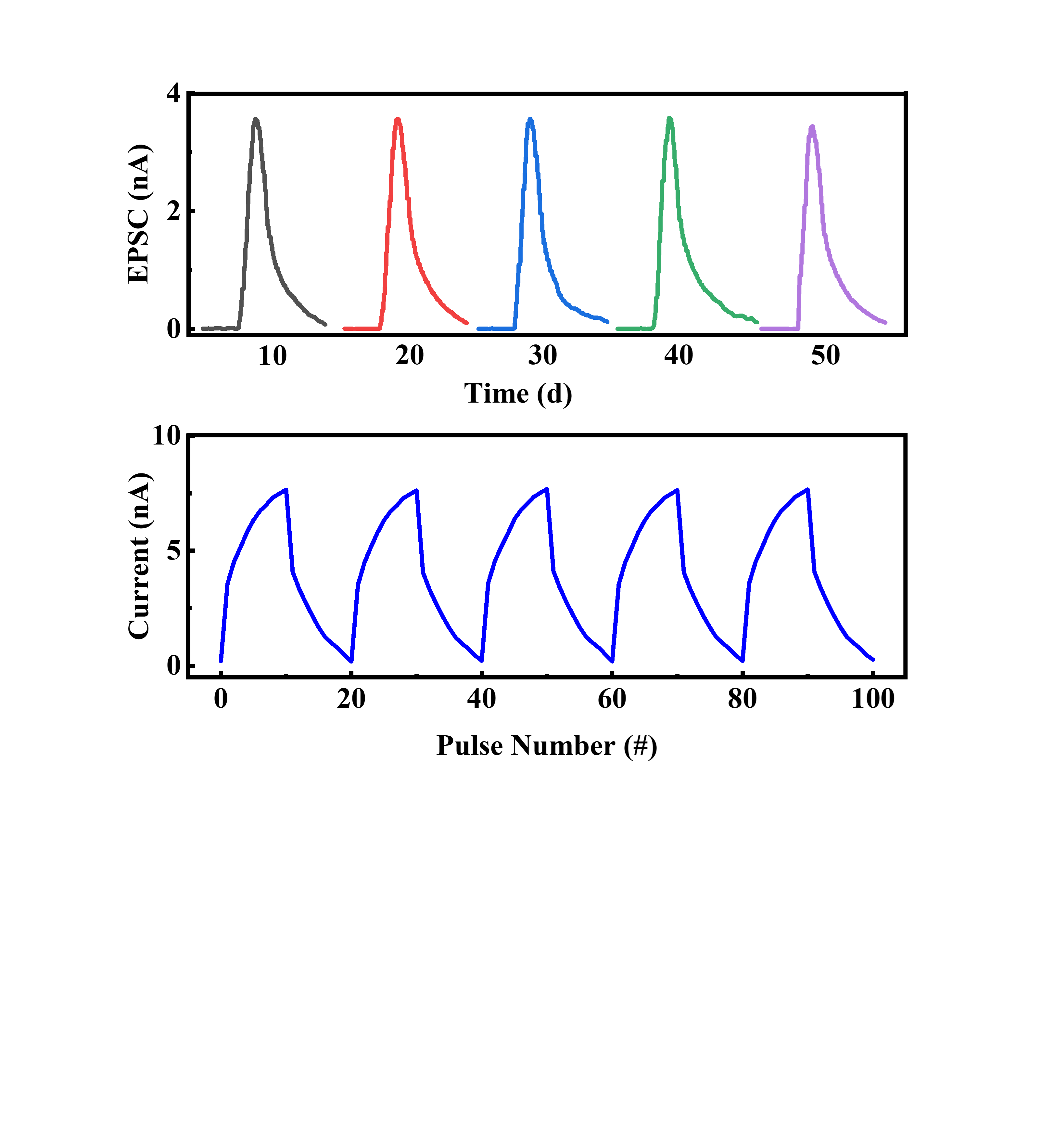


**Figure S7.** EPSC as a function of time for C60@GO optoelectronic synapse under 395 nm illumination (optical pulse of 1 s, 5 mW cm^-2^).

**
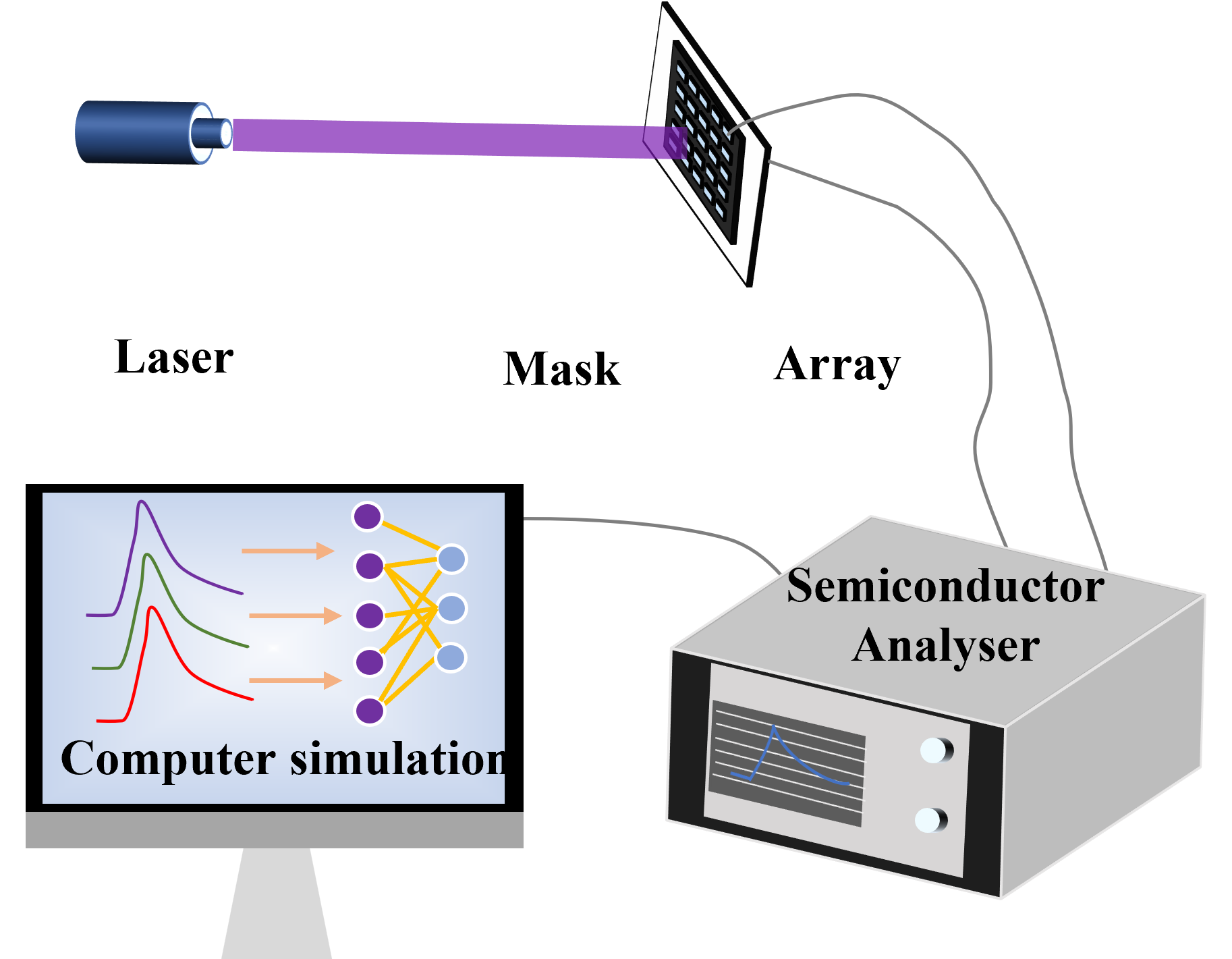
**

**Figure S8.** Experimental process roadmap——The beam is emitted from the laser directly onto the C60@GO array. The data is then collected via a semiconductor parameter instrument and subsequently collated and calculated by a computer.


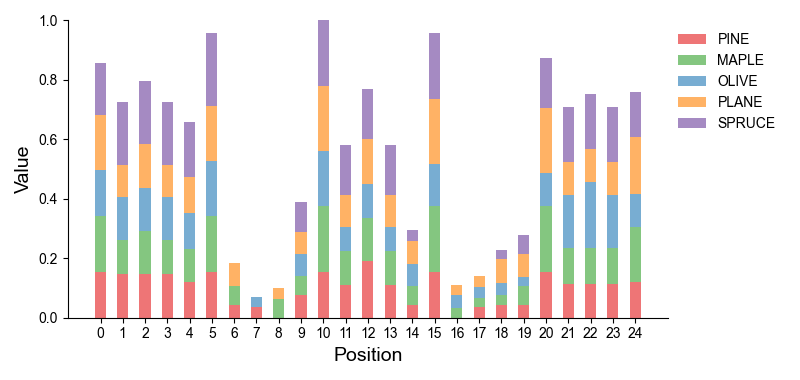


**Figure S9.** Feature vectors of the last frame (letter ‘E’) for 'PLANE,' 'OLIVE,' 'MAPLE,' 'PINE,' and 'SPRUCE'. The x-axis represents the pixel position index in the last frame, while the length of the color bar indicates the current intensity corresponding to each word at that position.
